# Antiphospholipid Antibodies and COVID‐19: A Systematic Review of Clinical Implications

**DOI:** 10.1002/iid3.70134

**Published:** 2025-02-03

**Authors:** Tahereh Sabaghian, Amir Behnam Kharazmi, Fatemeh Omidi, Bahareh Hajikhani, Shabnam Tehrani, Sayna Mardani, Amir Hashem Shahidi Bonjar, Rosella Centis, Lia D'Ambrosio, Giovanni Sotgiu, Fabio Angeli, Mohammad Javad Nasiri, Giovanni Battista Migliori

**Affiliations:** ^1^ Clinical Research Development Center Imam Hossein Educational Hospital, Shahid Beheshti University of Medical Sciences Tehran Iran; ^2^ Department of Internal Medicine School of Medicine, Imam Hossein Medical Center, Shahid Beheshti University of Medical Sciences Tehran Iran; ^3^ Department of Cardiology Imam Hossein Hospital, Shahid Beheshti University of Medical Sciences Tehran Iran; ^4^ Department of Microbiology School of Medicine, Shahid Beheshti University of Medical Sciences Tehran Iran; ^5^ Servizio di Epidemiologia Clinica delle Malattie Respiratorie Istituti Clinici Scientifici Maugeri IRCCS Tradate Italy; ^6^ Public Health Consulting Group Lugano Switzerland; ^7^ Clinical Epidemiology and Medical Statistics Unit, Department of Medicine, Surgery and Pharmacy University of Sassari Sassari Italy; ^8^ Department of Medicine and Cardiopulmonary Rehabilitation Maugeri Care and Research Institute, IRCCS Tradate Italy; ^9^ Department of Medicine and Technological Innovation (DiMIT) University of Insubria Varese Italy

**Keywords:** antiphospholipid antibodies, COVID‐19, SARS‐CoV‐2, systematic review, thrombosis

## Abstract

**Introduction:**

As the COVID‐19 pandemic transitions, understanding the intricate dynamics of the disease becomes paramount. This systematic review explores the role of antiphospholipid antibodies in COVID‐19, focusing on their potential clinical implications.

**Methods:**

This systematic review, following PRISMA guidelines, assesses studies exploring the link between antiphospholipid antibodies and COVID‐19. PubMed/Medline, Embase, and Scopus were searched for relevant studies published up to December 22, 2024. Inclusion criteria comprised studies involving patients diagnosed with COVID‐19 and reporting on the presence of antiphospholipid antibodies. The risk of bias in individual studies was evaluated using the Joanna Briggs Institute appraisal tool.

**Results:**

Our Study includes 59 records involving a total of 28,489 COVID‐19 patients. Antiphospholipid antibodies were tested in 14,498 COVID‐19 patients. It was observed that 50.84% of patients tested positive for antiphospholipid antibodies. Various types of antiphospholipid antibodies, including Anticardiolipin, Anti beta2 glycoproteins, and Lupus anticoagulant antibody, displayed prevalence rates in the patients with thrombosis. The overall frequency of antiphospholipid antibodies in thrombosis patients was 38.55%.

**Conclusion:**

The presence of antiphospholipid antibodies in a significant proportion of COVID‐19 patients underscores the need for a detailed investigation into their role in thrombotic events. Our study highlights potential avenues for targeted interventions. However, the evolving nature of COVID‐19 necessitates continued research efforts to clarify clinical implications and optimize management strategies in this complex landscape of thrombosis and immunology. The review reveals some limitations, such as variability in study designs and demographics and inherent differences in methodologies among included studies. Future studies should address these limitations with standardized methodologies for more conclusive findings.

## Graphical abstract

The emergence of antiphospholipid antibodies in a significant proportion of COVID‐19 patients highlights a crucial area of investigation in the realm of thrombotic events associated with the disease. Our study underscores the pressing need for a meticulous evaluation of these antibodies' involvement in coagulopathic manifestations, shedding light on potential avenues for targeted interventions. While the clinical implications of this relationship are promising, the evolving nature of COVID‐19 demands continued research efforts to solidify our understanding and pave the way for more effective management strategies in this intricate landscape of thrombosis and immunology.

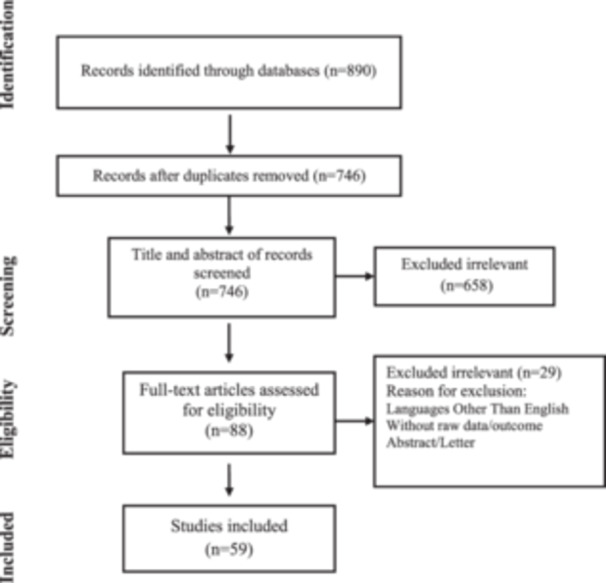

## 1 Introduction

As the COVID‐19 pandemic transitions into a new phase, marked by vaccination campaigns, public health measures, and lessons learned from its earlier stages [1], it offers an opportunity to reflect on the challenges it presented to the global healthcare community. The initial rapid spread of the novel coronavirus SARS‐CoV‐2 and its multifaceted impact on human health prompted an urgent need for comprehensive research efforts. In the quest to understand the complexities of this disease, we are exploring the role of antiphospholipid antibodies in COVID‐19 patients. Several studies have documented a heightened incidence of thrombosis among COVID‐19 patients, with varying but significant rates observed globally. Research indicates that up to 50% of hospitalized COVID‐19 patients may develop thrombotic complications, encompassing conditions such as deep vein thrombosis, pulmonary embolism, and arterial thrombosis. These complications not only increase morbidity and mortality rates but also pose substantial challenges in clinical management strategies [2, 3, 4, 5]. These findings have raised pertinent questions about the potential role of antiphospholipid antibodies in the pathophysiology of the disease.

Antiphospholipid antibodies represent a group of autoantibodies targeting phospholipid‐binding plasma proteins [6, 7, 8]. These antibodies are renowned for their association with hypercoagulable states, predisposing individuals to thrombotic events, such as deep vein thrombosis, pulmonary embolism, and stroke [9, 10, 11]. The presence of these antibodies in COVID‐19 patients has led to a surge in research dedicated to unraveling their implications in the context of the ongoing pandemic [12, 13].

This systematic review aims to provide a comprehensive analysis of the existing body of knowledge surrounding the role of antiphospholipid antibodies in COVID‐19.

## 2 Methods

### 2.1 Study Design

This systematic review follows the “Preferred Reporting Items for Systematic Reviews and Meta‐Analyses” (PRISMA) guidelines [14].

### 2.2 Search Strategy

Our search aimed to identify studies on the association between antiphospholipid antibodies and COVID‐19. We conducted thorough searches in PubMed/Medline, Embase, and Scopus, considering studies published up to December 22, 2024. The initial search date was not restricted. We implemented a search strategy that meticulously combined Mesh terms, Emree, and free text to ensure a thorough exploration of the literature on “antiphospholipid antibodies” and “COVID‐19.” Synonymous terms such as “lupus anticoagulant,” “antiphospholipid syndrome,” “coronavirus disease 2019,” and “SARS‐CoV‐2 infection” were also incorporated to broaden the scope of our search.

### 2.3 Study Selection

To ensure methodological rigor, we initiated our study selection process by merging records identified through systematic searches of PubMed/Medline, Embase, and Scopus, and then removing duplicates using EndNote X8 (Thomson Reuters, New York, NY, USA). Initial screening of titles and abstracts was independently conducted by two reviewers (TS and BH), with studies that did not meet the predefined inclusion criteria excluded at this stage. In cases of discrepancy, a third reviewer (MJN) intervened to achieve consensus.

Our inclusion criteria focused on studies involving populations diagnosed with COVID‐19 and assessing antiphospholipid antibodies. Specifically, we included cross‐sectional, case‐control, or cohort studies that reported findings related to antiphospholipid antibodies in COVID‐19 patients. The review was limited to studies published in the English language.

Exclusion criteria encompassed studies primarily focused on conditions other than COVID‐19, mortality studies, autopsy‐based investigations, and those lacking full‐text availability. Additionally, we excluded review articles, duplicate publications, conference abstracts, editorials, and postmortem studies to ensure the relevance and specificity of our systematic review.

### 2.4 Data Extraction

Data extraction followed a pre‐designed form and was performed by MJN and BH. Discrepancies were addressed through mutual agreement. Extracted information included the first author's name, study type, year of publication, study location, study population characteristics (age, gender, occupation), laboratory findings, patient's history, treatment patients received, and counts for various populations within the studies.

### 2.5 Risk of Bias in Individual Studies

The quality assessment of the included studies was conducted by two reviewers (ST and SM) using the Joanna Briggs Institute appraisal tool [15]. In case of any discrepancies, a third reviewer (MJN) was involved.

Various methodological features of the studies were assessed using this tool, including participant recruitment process, study setting, outcome measurement, and statistical analysis. Studies with a total score of > 7 were considered to have low risk of bias.

## 3 Results

### 3.1 Study Characteristics

**Figure 1:**
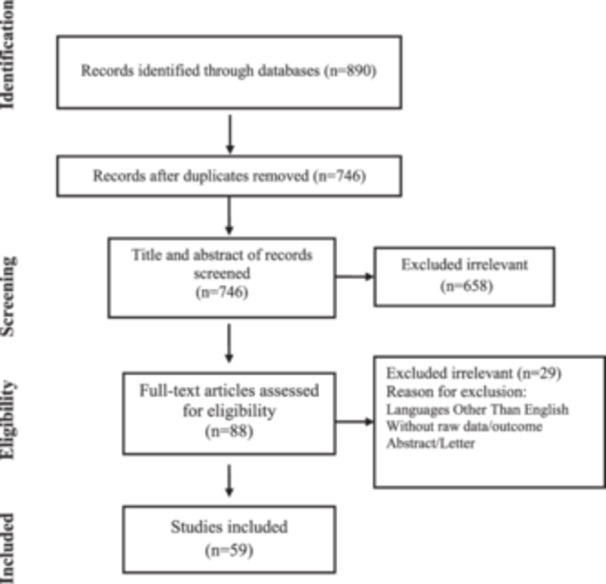
Flow chart of study selection for inclusion in the systematic review.

A total of 59 studies, involving a total of 28,489 COVID‐19 patients, were included in this systematic review [16, 17, 18, 19, 20, 21, 22, 23, 24, 25, 26, 27, 28, 29, 30, 31, 32, 33, 34, 35, 36, 37, 38, 39, 40, 41, 42, 43, 44, 45, 46, 47, 48, 49, 50, 51, 52, 53, 54, 55, 56, 57, 58, 59, 60, 61, 62, 63, 64, 65, 66, 67, 68, 69, 70, 71, 72, 73, 74] (Figure 1). These studies encompassed a variety of research designs, including case reports, cross‐sectional, cohorts, and case‐control studies. Geographically, they spanned several countries, such as the United States, Italy, France, China, Spain, and others, contributing to the global understanding of antiphospholipid antibodies in COVID‐19. The average age of patients across the 59 studies was 57.67 years, reflecting the wide age range of COVID‐19 cases investigated (Table 1). This demonstrates that individuals of various age groups have been affected by COVID‐19. Additionally, the average body mass index (BMI), as reported in 10 studies, was 28.45, suggesting a diverse range of BMIs among patients.

**Table 1:** Basic characteristics of included studies.

References	Year	Study design	Country	Number of patients
Ren et al. [74]	2024	Case report	China	1
Li et al. [72]	2024	Case report	China	1
Capozzi et al. [71]	2023	Case series	Italy	10
Pan et al. [73]	2023	Cohort	China	39
Karahan et al. [16]	2022	Cohort	Turkey	31
Shah et al. [17]	2022	Case‐control	USA	40
Bnina et al. [18]	2022	Cohort	Tunisia	54
Kahlon et al. [19]	2022	Cohort	USA	28
Zeng et al. [20]	2022	Case series	China	87
Demoulin et al. [21]	2022	Case report	France	1
Noordermeer et al. [22]	2022	Cohort	Netherland	169
Mendel et al. [23]	2022	Cohort	Canada	289
Espinosa et al. [24]	2022	Cohort	Spain	158
Shams Aldeen et al. [25]	2022	Case‐control	Sudan	45
Cuadros Sánchez et al. [26]	2022	Case report	Spain	1
Ammous et al. [27]	2021	Case reports	USA	1
Balanchivadze et al. [28]	2021	Case reports	USA	2
Cristiano et al. [29]	2021	Cohort	Italia	92
Hamade et al. [30]	2021	Cohort	France	41
Loos et al. [31]	2021	Case report	Belgium	1
Le Joncour et al. [32]	2021	Cohort	France	104
Pascolini et al. [33]	2021	Case‐control	Italy	33
Showers et al. [34]	2021	Case reports	USA	1
Hollerbach et al. [35]	2021	Cohort	Germany	174
Gendron et al. [36]	2021	Cohort	France	154
Roncati, Corsi, and Barbolini [37]	2021	Case report	Italy	1
Constans et al. [38]	2021	Cohort	Spain	211
Rothstein et al. [39]	2020	Cohort	USA	20
Mullaguri et al. [40]	2020	Case series	USA	3
Valencia Manrique, Ghosh, and Boma [41]	2020	Case reports	USA	1
Vollmer et al. [42]	2020	Case series	France	79
Alharthy et al. [43]	2020	Case series	Saudia Arabia	3
Amezcua‐Guerra et al. [44]	2020	Case series	Mexico	21
Bamgboje et al. [45]	2020	Case report	USA	1
Devreese et al. [46]	2020	Cohort	Belgium	31
Fan et al. [47]	2020	Cohort	China	86
Ferrari et al. [48]	2020	Cohort	France	89
Galeano‐Valle et al. [49]	2020	Cohort	Spain	24
Gemciogju et al. [50]	2020	Case report	Turkey	1
Goldberg et al. [51]	2020	Case report	USA	1
de Ocáriz et al. [52]	2020	Cohort	Spain	27
Hossri et al. [53]	2020	Case report	USA	2
Iguina et al. [54]	2020	Case report	USA	1
Injean et al. [55]	2020	Cohort	USA	71
Jizzini, Shah, and Zhou [56]	2020	Case report	USA	1
Mantovani Cardoso et al. [57]	2020	Case report	USA	1
Renaud‐Picard et al. [58]	2020	Case report	France	1
Reyes Gil et al. [59]	2020	Cohort	USA	68
Shoskes et al. [60]	2020	Case report	USA	1
Siguret et al. [61]	2020	Cohort	France	74
Sung and Anjum [62]	2020	Case report	USA	1
Upson et al. [63]	2020	Case report	USA	1
Vlachoyiannopoulos et al. [64]	2020	Case series	Greece	29
Xiao et al. [65]	2020	Case series	China	79
Yarlagadda et al. [66]	2020	Case report	USA	1
Zayet et al. [67]	2020	Case report	France	2
Zhang et al. [68]	2020	Case series	China	19
Previtali et al. [69]	2020	Case series	Italy	35
Zhang et al. [70]	2020	Case report	China	3

### 3.2 Risk of Bias Assessment

The results of the critical appraisal of included studies were summarized in Supporting Information S1 (Tables S1–S4). Included studies were identified as having a low risk of bias.

### 3.3 Patient History

Several variables related to patient history were assessed (Table 2). Notably, 25.69% of COVID‐19 patients had a history of thrombosis, indicating that this cohort had a higher propensity for thrombotic events. Gender distribution revealed that 60.22% of the total sample were male, and 40.04% were female. These findings underline the gender diversity among COVID‐19 patients.

**Table 2:** Patient history.

Variable	No. of study	*n*/*N*	%
Number of patients with thrombosis	49	512/1993	25.69
Male of the total sample	39	1399/2323	60.22
Male with thrombosis	27	175/668	26.20
Male with antiphospholipid antibodies	16	234/830	28.19
Female of the total sample	35	911/2275	40.04
Female with thrombosis	29	180/874	20.59
Female with antiphospholipid antibodies	16	192/1038	18.50
Smoking	7	52/738	7.05
History of thrombosis	13	136/1014	13.37
History of cancer	11	106/837	12.66
Active cancer	5	28/189	14.81
Ischemic heart disease	17	227/1214	18.70
Congestive heart failure	5	37/55	67.27
Stroke	14	98/859	11.41
Peripheral artery disease	6	20/178	11.24
Hypertension	29	741/1740	42.59
Dyslipidemia	14	225/1219	18.46
Diabetes mellitus	26	422/1731	24.38
Pulmonary disease	12	103/855	12.05
Chronic kidney disease	12	73/762	9.58
Platelet inhibitor	7	77/366	21.04
Oral anticoagulant	7	74/740	10.00
Fatigue and myalgia	13	201/357	56.30
Dyspnea	23	459/799	57.45
Cough	24	513/865	59.31
Fever	25	522/835	62.51
Diarrhea	8	46/244	18.85
Thoracic pain	4	23/108	21.30
Anosmia	3	13/109	11.93
Atrial fibrillation	5	17/303	5.61
Obesity	4	18/85	21.18

*Note: n*, the number of patients with any variables; *N*, the total number of studied patients.

### 3.4 Prognosis of Antiphospholipid Antibodies

The review investigated several variables related to patient prognosis (Table 3). Antiphospholipid antibodies were tested in 14,498 COVID‐19 patients. It was observed that 46.18% of patients tested negative for antiphospholipid antibodies antiphospholipid antibodies, while 50.84% tested positive for antiphospholipid antibodies. Additionally, outcomes, such as the need for intensive care unit (ICU) admission, intubation, and hospital mortality, were explored. For instance, 19.65% of patients without antiphospholipid antibodies required ICU admission, compared to 35.17% of those with antiphospholipid antibodies.

**Table 3:** Prognosis of antiphospholipid antibodies.

Variable	No. of study	*n*/*N*	%
Negative antiphospholipid antibodies	32	1173/2540	46.18
Positive antiphospholipid antibodies	55	1302/2561	50.84
Nasal O_2_, without antiphospholipid antibodies	3	137/158	86.71
Nasal O_2_, with antiphospholipid antibodies	7	7/7	100
ICU, without antiphospholipid antibodies	17	233/1186	19.65
ICU, with antiphospholipid antibodies	34	567/1612	35.17
Intubation, without antiphospholipid antibodies	11	140/953	14.69
Intubation, with antiphospholipid antibodies	25	305/1032	29.55
Hospital mortality, without antiphospholipid antibodies	17	163/1281	12.72
Hospital mortality, with antiphospholipid antibodies	24	183/1310	13.97
Discharge, without antiphospholipid antibodies	22	518/1278	40.53
Discharge, with antiphospholipid antibodies	28	463/1309	35.37

*Note: n*, the number of patients with any variables; *N*, the total number of studied patients

### 3.5 Treatment

Treatment‐related variables were also examined in the review (Table 4). A significant proportion of patients, 55.83%, received heparin or enoxaparin. This highlights the widespread use of anticoagulation therapy in the management of COVID‐19. Renal replacement therapy, thrombolysis, and other interventions were also assessed, although they were less prevalent in the included studies.

**Table 4:** Treatment history.

Variable	No. of study	*n*/*N*	%
Heparin	40	986/1766	55.83
Renal replacement therapy	6	70/317	22.08
Thrombolysis	4	5/25	20

*Note: n*, the number of patients with any variables; *N*, the total number of studied patients

### 3.6 Laboratory Findings of Antiphospholipid Antibodies

Various types of antiphospholipid antibodies, including Anticardiolipin (IgG, IgM, IgA), Anti beta2 glycoproteins (IgG, IgM), and Lupus anticoagulant antibody, displayed prevalence rates in the examined patients with thrombosis (Table 5). Specifically, Anticardiolipin IgG exhibited a prevalence of 11.70%, Anticardiolipin IgM had a prevalence of 16.50%, and Anticardiolipin IgA showed the highest prevalence at 21.15%. For Anti beta2 glycoproteins, the prevalence of IgG was 3.57%, and IgM had a prevalence of 7.10%. Lupus anticoagulant antibody demonstrated a prevalence of 17.20%. Notably, the overall frequency of positive antiphospholipid antibodies in thrombosis patients was 38.55%.

**Table 5:** Laboratory findings of antiphospholipid antibodies.

Variable	No. of study	*n*/*N*	%
Anticardiolipin IgG with thrombosis	24	153/1308	11.70
Anticardiolipin without thrombosis	6	37/469	7.89
Anticardiolipin IgM with thrombosis	30	231/1400	16.50
Anticardiolipin IgM without thrombosis	9	41/580	7.07
Anticardiolipin IgA with thrombosis	12	70/331	21.15
Anti beta2 glycoproteins IgG with thrombosis	10	35/981	3.57
Anti beta2 glycoproteins IgG without thrombosis	5	55/429	12.59
Anti beta2 glycoproteins IgM with thrombosis	15	80/1127	7.10
Anti beta2 glycoproteins IgM without thrombosis	5	44/366	12.02
Lupus anticoagulant antibody with thrombosis	28	320/1860	17.20
Lupus anticoagulant antibody without thrombosis	12	299/998	29.96
Positive antiphospholipid antibody total with thrombosis	24	355/921	38.55
Positive antiphospholipid antibody total without thrombosis	10	207/560	36.96

*Note: n*, the number of patients with any variables; *N*, the total number of studied patients.

## 4 Discussion

The systematic review presented here synthesizes and analyzes the existing body of literature regarding the role of antiphospholipid antibodies in COVID‐19. The association between antiphospholipid antibodies and COVID‐19 has emerged as a significant area of investigation, given the multifaceted nature of this global pandemic.

Our review indicates that understanding the interplay between antiphospholipid antibodies and COVID‐19 is of paramount importance. Several key findings and implications can be drawn from the collective data analyzed in this study.

### 4.1 Implications of Antiphospholipid Antibodies in COVID‐19

Our analysis reveals a substantial body of evidence documenting the presence of antiphospholipid antibodies in COVID‐19 patients. These autoantibodies, known for their association with hypercoagulation and thrombotic events, have garnered considerable attention during the pandemic. The frequency of antiphospholipid antibodies in COVID‐19 patients underscores the need to consider their potential implications in disease pathophysiology. Antiphospholipid antibodies have also significant clinical implications both in general patients and specifically in those with COVID‐19, particularly due to their association with thrombotic events. In general patients, antiphospholipid antibodies are known to predispose individuals to a spectrum of thrombotic complications, including venous and arterial thrombosis. These antibodies interfere with the body's normal regulation of blood clotting, leading to an increased risk of deep vein thrombosis, pulmonary embolism, stroke, and other thrombotic events. Moreover, antiphospholipid antibodies are also associated with recurrent pregnancy loss and other obstetric complications, further highlighting their diverse clinical impact. In the context of COVID‐19, the presence of antiphospholipid antibodies has been increasingly recognized as a potential contributor to the hypercoagulable state observed in severe cases. COVID‐19 itself is associated with a high incidence of thrombotic complications, and patients with pre‐existing antiphospholipid antibodies may be at even greater risk. Studies have reported that COVID‐19 patients with antiphospholipid antibodies are more likely to experience severe thrombotic events, such as pulmonary embolism and microvascular thrombosis, which can significantly worsen clinical outcomes and increase mortality rates. Understanding the impact of antiphospholipid antibodies in both general patient populations and specifically in COVID‐19 patients underscores the importance of early detection and appropriate management strategies. Clinicians managing COVID‐19 patients should be vigilant for signs of thrombosis in individuals with known antiphospholipid antibodies, and proactive measures such as anticoagulation therapy may be warranted to mitigate these risks and improve patient outcomes.

### 4.2 Thrombotic Events in COVID‐19

The emergence of thrombotic events in COVID‐19 patients has been a significant concern. Many studies have reported a higher incidence of thrombosis in individuals with COVID‐19, raising pertinent questions about the potential involvement of antiphospholipid antibodies in these coagulopathic manifestations [75, 76, 77]. Studies have reported that up to 50% of hospitalized COVID‐19 patients experience thrombotic events [2, 3, 4, 5]. Historically, antiphospholipid antibodies have been linked to autoimmune conditions and diseases characterized by a heightened risk of thrombosis. This connection has prompted a critical examination of whether these autoantibodies contribute to the thrombotic complications observed in COVID‐19.

### 4.3 Long‐Term Events in aPL‐Positive Patients

The long‐term effects of antiphospholipid antibodies in COVID‐19 survivors are becoming increasingly evident, particularly as more than 4 years have passed since the onset of the pandemic. Recent studies have highlighted a notable association between antiphospholipid antibodies and post‐acute sequelae of SARS‐CoV‐2 (PASC), a condition encompassing a range of persistent symptoms and complications following acute infection. Su et al. [78] demonstrated that a subset of PASC patients tested positive for various autoantibodies, including antiphospholipid antibodies, suggesting a prolonged autoimmune response. Moreover, micro‐thromboembolic complications have been observed in patients recovering from COVID‐19, further implicating antiphospholipid antibodies in long‐lasting cardiovascular risks [79]. These findings underline the necessity of long‐term monitoring for thrombotic and autoimmune sequelae in aPL‐positive patients. The potential for chronic complications, including increased risks of cardiovascular diseases, highlights the importance of developing targeted management strategies to address the enduring impact of these antibodies in COVID‐19 survivors.

### 4.4 Clinical Implications

The possible clinical implications of this review are significant in several ways. First, it underscores the importance of screening for antiphospholipid antibodies in COVID‐19 patients, particularly those at higher risk for thrombotic events. Early detection can aid in identifying individuals who may benefit from targeted interventions such as anticoagulation therapy, potentially reducing the incidence of severe complications like pulmonary embolism or stroke. Moreover, understanding the association between antiphospholipid antibodies and COVID‐19 thrombosis may guide clinicians in tailoring treatment strategies and improving patient outcomes through more personalized care approaches. Further research in this area could refine diagnostic criteria and therapeutic protocols, ultimately enhancing the management of COVID‐19 patients with underlying thrombotic risks.

### 4.5 Limitations and Future Directions

As with any systematic review, there are limitations to consider. The diversity of study designs, populations, and methodologies among included studies may introduce inherent limitations related to comparability and generalizability. Variations in participant characteristics, healthcare settings, and geographic locations could influence the consistency of findings across different studies. Therefore, caution should be exercised when interpreting the collective evidence, and future research should strive for more standardized approaches to enhance the reliability and applicability of findings in clinical practice. Future research should continue to investigate the role of antiphospholipid antibodies in COVID‐19, particularly focusing on well‐designed, high‐quality studies. Prospective studies with larger sample sizes and standardized methodologies could provide further insights into the prevalence and implications of these antibodies.

## 5 Conclusion

In conclusion, the relationship between antiphospholipid antibodies and COVID‐19 is a complex and evolving field. While it has the potential to impact diagnostic and therapeutic strategies, further research is required to provide more conclusive insights. The lessons learned from this systematic review should guide future investigations and contribute to a more comprehensive understanding of this intriguing area of study.

## Author Contributions

All authors contributed equally to the conception, design, data collection, analysis, interpretation, and writing of the manuscript.

## Ethics Statement

The authors have nothing to report.

## Consent

All authors have consented to the publication of this manuscript.

## Conflicts of Interest

The authors declare no conflicts of interest.

## Supporting information

Supporting information.

Supporting information.

## Data Availability

All data and materials relevant to this study are included within the manuscript.
