# Supplementary material for: Antiphospholipid Antibodies and COVID‐19: A Systematic Review of Clinical Implications
Source: Immun Inflamm Dis. 2025 Feb 3;13(2):e70134. doi: 10.1002/iid3.70134 (PMC11789270; doi:10.1002/iid3.70134)
Supplement: Supplementary file 1 — Supporting information. [file IID3-13-e70134-s001.docx]

Table S1. Quality assessment of cohort studies

| Author | 1 | 2 | 3 | 4 | 5 | 6 | 7 | 8 | 9 | 10 | 11 |
| --- | --- | --- | --- | --- | --- | --- | --- | --- | --- | --- | --- |
| Pan | Yes | Yes | Yes | Unclear | Unclear | Unclear | Yes | Unclear | Unclear | Unclear | Yes |
| Karahan | Yes | Yes | Yes | Unclear | Unclear | Unclear | Yes | Unclear | Unclear | Unclear | Yes |
| Bnina | Yes | Yes | Yes | Unclear | Unclear | Unclear | Yes | Unclear | Unclear | Unclear | Yes |
| Kahlon | Yes | Yes | Yes | Unclear | Unclear | Unclear | Yes | Unclear | Unclear | Unclear | Yes |
| [Noordermeer](https://onlinelibrary.wiley.com/authored-by/ContribAuthorRaw/Noordermeer/Tessa) | Yes | Yes | Yes | Unclear | Unclear | Unclear | Yes | Unclear | Unclear | Unclear | Yes |
| Mendel | Yes | Yes | Yes | Unclear | Unclear | Unclear | Yes | Unclear | Unclear | Unclear | Yes |
| Spinosa | Yes | Yes | Yes | Unclear | Unclear | Unclear | Yes | Unclear | Unclear | Unclear | Yes |
| Cristiano | Yes | Yes | Yes | Unclear | Unclear | Unclear | Yes | Unclear | Unclear | Unclear | Yes |
| Hamade | Yes | Yes | Yes | Unclear | Unclear | Unclear | Yes | Unclear | Unclear | Unclear | Yes |
| Le Joncour | Yes | Yes | Yes | Unclear | Unclear | Unclear | Yes | Unclear | Unclear | Unclear | Yes |
| Hollerbach | Yes | Yes | Yes | Unclear | Unclear | Unclear | Yes | Unclear | Unclear | Unclear | Yes |
| Gendron | Yes | Yes | Yes | Unclear | Unclear | Unclear | Yes | Unclear | Unclear | Unclear | Yes |
| [Constans](https://www.ncbi.nlm.nih.gov/pubmed/?term=Constans%20M%5BAuthor%5D&cauthor=true&cauthor_uid=34743034) | Yes | Yes | Yes | Unclear | Unclear | Unclear | Yes | Unclear | Unclear | Unclear | Yes |
| Rothstein | Yes | Yes | Yes | Unclear | Unclear | Unclear | Yes | Unclear | Unclear | Unclear | Yes |
| Devreese | Yes | Yes | Yes | Unclear | Unclear | Unclear | Yes | Unclear | Unclear | Unclear | Yes |
| Fan | Yes | Yes | Yes | Unclear | Unclear | Unclear | Yes | Unclear | Unclear | Unclear | Yes |
| Ferrari | Yes | Yes | Yes | Unclear | Unclear | Unclear | Yes | Unclear | Unclear | Unclear | Yes |
| Valle | Yes | Yes | Yes | Unclear | Unclear | Unclear | Yes | Unclear | Unclear | Unclear | Yes |
| de Ocáriz | Yes | Yes | Yes | Unclear | Unclear | Unclear | Yes | Unclear | Unclear | Unclear | Yes |
| Injean | Yes | Yes | Yes | Unclear | Unclear | Unclear | Yes | Unclear | Unclear | Unclear | Yes |
| Gil | Yes | Yes | Yes | Unclear | Unclear | Unclear | Yes | Unclear | Unclear | Unclear | Yes |
| Siguret | Yes | Yes | Yes | Unclear | Unclear | Unclear | Yes | Unclear | Unclear | Unclear | Yes |

1. Were the two groups similar and recruited from the same population? 2. Were the exposures measured similarly to assign people to both exposed and unexposed groups? 3. Was the exposure measured in a valid and reliable way? 4. Were confounding factors identified? 5. Were strategies to deal with confounding factors stated? 6. Were the groups/participants free of the outcome at the start of the study (or at the moment of exposure)? 7. Were the outcomes measured in a valid and reliable way? 8. Was the follow up time reported and sufficient to be long enough for outcomes to occur? 9. Was follow up complete, and if not, were the reasons to loss to follow up described and explored? 10. Were strategies to address incomplete follow up utilized? 11. Was appropriate statistical analysis used?

Table S2. Quality assessment of case report studies

| Author | 1 | 2 | 3 | 4 | 5 | 6 | 7 | 8 |
| --- | --- | --- | --- | --- | --- | --- | --- | --- |
| Ren | Yes | Yes | Yes | Yes | Yes | Unclear | Unclear | Yes |
| Li Q | Yes | Yes | Yes | Yes | Yes | Unclear | Unclear | Yes |
| Demoulin | Yes | Yes | Yes | Yes | Yes | Unclear | Unclear | Yes |
| [Sánchez](https://www.tandfonline.com/author/Cuadros+S%C3%A1nchez%2C+Carlos) | Yes | Yes | Yes | Yes | Yes | Unclear | Unclear | Yes |
| Ammous | Yes | Yes | Yes | Yes | Yes | Unclear | Unclear | Yes |
| Balanchivadze | Yes | Yes | Yes | Yes | Yes | Unclear | Unclear | Yes |
| Loos | Yes | Yes | Yes | Yes | Yes | Unclear | Unclear | Yes |
| Showers | Yes | Yes | Yes | Yes | Yes | Unclear | Unclear | Yes |
| Roncati | Yes | Yes | Yes | Yes | Yes | Unclear | Unclear | Yes |
| Manrique | Yes | Yes | Yes | Yes | Yes | Unclear | Unclear | Yes |
| Bamgboje | Yes | Yes | Yes | Yes | Yes | Unclear | Unclear | Yes |
| Gemciogju | Yes | Yes | Yes | Yes | Yes | Unclear | Unclear | Yes |
| Goldberg | Yes | Yes | Yes | Yes | Yes | Unclear | Unclear | Yes |
| Hossri | Yes | Yes | Yes | Yes | Yes | Unclear | Unclear | Yes |
| Iguina | Yes | Yes | Yes | Yes | Yes | Unclear | Unclear | Yes |
| Jizzini | Yes | Yes | Yes | Yes | Yes | Unclear | Unclear | Yes |
| Cardoso | Yes | Yes | Yes | Yes | Yes | Unclear | Unclear | Yes |
| Picard | Yes | Yes | Yes | Yes | Yes | Unclear | Unclear | Yes |
| Shoskes | Yes | Yes | Yes | Yes | Yes | Unclear | Unclear | Yes |
| Sung | Yes | Yes | Yes | Yes | Yes | Unclear | Unclear | Yes |
| Upson | Yes | Yes | Yes | Yes | Yes | Unclear | Unclear | Yes |
| Yarlagadda | Yes | Yes | Yes | Yes | Yes | Unclear | Unclear | Yes |
| Zayet | Yes | Yes | Yes | Yes | Yes | Unclear | Unclear | Yes |
| Zhang | Yes | Yes | Yes | Yes | Yes | Unclear | Unclear | Yes |

1. Were patient’s demographic characteristics clearly described? 2. Was the patient’s history clearly described and presented as a timeline? 3. Was the current clinical condition of the patient on presentation clearly described? 4. Were diagnostic tests or assessment methods and the results clearly described? 5. Was the intervention(s) or treatment procedure(s) clearly described? 6. Was the post-intervention clinical condition clearly described? 7. Were adverse events (harms) or unanticipated events identified and described? 8. Does the case report provide takeaway lessons?

Table S3. Quality assessment of case series studies

| Author | 1 | 2 | 3 | 4 | 5 | 6 | 7 | 8 | 9 | 10 |
| --- | --- | --- | --- | --- | --- | --- | --- | --- | --- | --- |
| Capozzi | Yes | Yes | Yes | Yes | Yes | Yes | Yes | Yes | Unclear | Yes |
| [Zeng](https://pubmed.ncbi.nlm.nih.gov/?term=Zeng%20H%5BAuthor%5D) | Yes | Yes | Yes | Yes | Yes | Yes | Yes | Yes | Unclear | Yes |
| Mullaguri | Yes | Yes | Yes | Yes | Yes | Yes | Yes | Yes | Unclear | Yes |
| Voltmer | Yes | Yes | Yes | Yes | Yes | Yes | Yes | Yes | Unclear | Yes |
| Alharthy | Yes | Yes | Yes | Yes | Yes | Yes | Yes | Yes | Unclear | Yes |
| Guerra | Yes | Yes | Yes | Yes | Yes | Yes | Yes | Yes | Unclear | Yes |
| Vlachoyiannopoulos | Yes | Yes | Yes | Yes | Yes | Yes | Yes | Yes | Unclear | Yes |
| Xiao | Yes | Yes | Yes | Yes | Yes | Yes | Yes | Yes | Unclear | Yes |
| Zhang | Yes | Yes | Yes | Yes | Yes | Yes | Yes | Yes | Unclear | Yes |
| Previtali | Yes | Yes | Yes | Yes | Yes | Yes | Yes | Yes | Unclear | Yes |

1. Were there clear criteria for inclusion in the case series? 2. Was the condition measured in a standard, reliable way for all participants included in the case series? 3. Were valid methods used for identification of the condition for all participants included in the case series? 4. Did the case series have consecutive inclusion of participants? 5. Did the case series have complete inclusion of participants? 6. Was there clear reporting of the demographics of the participants in the study? 7. Was there clear reporting of clinical information of the participants? 8. Were the outcomes or follow up results of cases clearly reported? 9. Was there clear reporting of the presenting site(s)/clinic(s) demographic information? 10. Was statistical analysis appropriate?

Table S4. Quality assessment of case-control studies

| Author | 1 | 2 | 3 | 4 | 5 | 6 | 7 | 8 | 9 | 10 |
| --- | --- | --- | --- | --- | --- | --- | --- | --- | --- | --- |
| Shah | Yes | Unclear | Yes | Yes | Yes | Unclear | Unclear | Yes | Unclear | Yes |
| Aldeen | Yes | Unclear | Yes | Yes | Yes | Unclear | Unclear | Yes | Unclear | Yes |
| Pascolini | Yes | Unclear | Yes | Yes | Yes | Unclear | Unclear | Yes | Unclear | Yes |

1. Were the groups comparable other than the presence of disease in cases or the absence of disease in controls? 2. Were cases and controls matched appropriately? 3. Were the same criteria used for identification of cases and controls? 4. Was exposure measured in a standard, valid and reliable way? 5. Was exposure measured in the same way for cases and controls? 6. Were confounding factors identified? 7. Were strategies to deal with confounding factors stated? 8. Were outcomes assessed in a standard, valid and reliable way for cases and controls? 9. Was the exposure period of interest long enough to be meaningful? 10. Was appropriate statistical analysis used?
